# Fatigue patterns surrounding biologic disease-modifying antirheumatic drug injection in patients with an inflammatory rheumatic disease: an ecological momentary assessment study

**DOI:** 10.1007/s00296-024-05779-y

**Published:** 2025-01-11

**Authors:** Jette A. van Lint, Johanna E. Vriezekolk, Naomi T. Jessurun, Alfons A. den Broeder, Bart J. F. van den Bemt, Victor J. B. Huiskes

**Affiliations:** 1 Pharmacy, Radboudumc, Nijmegen, NL Netherlands; 2 Sint Maartenskliniek, Nijmegen, NL Netherlands; 3 Netherlands Pharmacovigilance Centre Lareb, Goudsbloemvallei 7, ’s-Hertogenbosch, 5237 MH Netherlands

**Keywords:** Surveys and questionnaires, Fatigue, bDMARD, Biologics, Rheumatic diseases, Self-assessment, Ecological momentary assessments

## Abstract

This study investigated severity, course and patterns of fatigue surrounding subcutaneous biological disease-modifying antirheumatic drug (bDMARD) injection in inflammatory rheumatic disease (IRD) patients using ecological momentary assessments and investigated self-reported adverse drug reactions (ADRs). In this prospective cohort study, IRD patients completed fatigue severity numeric rating scales (0–10) in web-based ecological momentary assessments in three waves of five days surrounding bDMARD injection. The course of fatigue was measured by the change in fatigue from pre-dosing to post-dosing scores and was classified as: worsening, improving or no clinically relevant change. A pattern was defined as a course of worsening, improving or no clinically relevant change in fatigue in at least two out of three waves for patients completing assessments across all three waves. ADRs could be reported on day five of each wave. In total 609 participants completed ecological momentary assessments surrounding 1541 bDMARD injections. Overall average fatigue severity across all three waves was 4.5 (± SD 2.4) and 78% experienced severe fatigue in at least one assessment. Of 398 patients completing all three waves, 61% had no clinically relevant change in fatigue in at least two out of three waves, 13% had a pattern of worsening fatigue and 18% had a pattern of improving fatigue. Of 398 patients, 36% had a consistent pattern in all three waves. IRD patients using a bDMARD may consistently experience specific fatigue patterns surrounding bDMARD administration. These patterns provide insights for clinical practice and could be used to inform patients properly.

**Supplementary Information:**

The online version contains supplementary material available at 10.1007/s00296-024-05779-y.

## Introduction

Fatigue is an important complaint in patients with inflammatory rheumatic diseases (IRDs) with half of the IRD patients experiencing severe fatigue, greatly impacting their quality of life [1, 2]. Fatigue can occur with an overwhelming intensity impacting daily lives on physical, cognitive, emotional and social level [3–5]. Fatigue is associated with increased healthcare use and considerable societal costs as a consequence [6]. While biological, physiological and psychosocial mechanisms have been implicated in fatigue, its exact pathogenesis remains unclear and the causes of fatigue are considered multifactorial [7, 8]. Prevalent fatigue is only weakly associated with disease activity and strongly associated with pain, poor sleep, obesity, lower aerobic condition and depression [4, 9]. So in general, especially in patients with low disease activity or remission, fatigue seems unrelated to the IRD.

Interestingly, fatigue has been reported and is labelled as an adverse drug reaction (ADR) of conventional and biological disease-modifying antirheumatic drugs, which are effective and essential therapeutic options for treating IRDs as recommended by the European Alliance of Associations for Rheumatology [9–14]. The relation between fatigue and bDMARDs appears contradictory. A review of 32 studies has demonstrated some improvement in fatigue in active rheumatoid arthritis (RA) following bDMARD treatment [15]. In contrast, some patients self-reported fatigue as an ADR of biologics in a cohort event monitoring study [16]. Out of 1,382 patients in this study, 8% spontaneously mentioned fatigue as an ADR with half of them (4% of 1,382 patients) describing it as a postdosing reaction. These patients experienced post-injection fatigue, often occurring within one day following injection, which typically resolved within a week but recurred after subsequent biologic injections. Although a pharmacological base for fatigue caused by bDMARDs seems unlikely, it is of interest to study this frequently mentioned complaint.

To date, fatigue fluctuations around bDMARD injection and postdosing fatigue shortly after injection have not been systematically described and, even though various treatment options for fatigue have been investigated, management of fatigue in IRD patients in general remains challenging and requires a tailored approach [9, 17]. Prospectively measuring the course of fatigue surrounding bDMARD injections as experienced by patients may substantiate and improve understanding and management of fatigue in IRDs. Therefore, this explorative study aimed to investigate and describe the severity and course of fatigue surrounding subcutaneous bDMARD injection in IRD patients using ecological momentary assessments and to describe self-reported adverse drug reactions (ADR) after bDMARD injection.

## Methods

### Study design

This prospective cohort study utilized the fatigue severity numeric rating scale (NRS), the primary outcome, in web-based ecological momentary assessments to investigate fatigue severity, the course of fatigue surrounding an injection and patterns in the course of fatigue surrounding multiple injections in patients with IRDs. Self-reported ADRs were investigated as a secondary outcome. Ecological momentary assessments were scheduled in three waves of five days surrounding a bDMARD injection to assess intra-individual variation in patterns in the course of fatigue surrounding bDMARD injections.

### Setting and participants

IRD patients using a subcutaneously administered bDMARD prescribed by a physician in the Sint Maartenskliniek, a specialised rheumatic disease centre in the Netherlands, were invited to participate in this study. Inclusion criteria were: 18 years or older and an active prescription for one of the following subcutaneous bDMARDs: abatacept, adalimumab, certolizumab pegol, etanercept, sarilumab or tocilizumab for a clinical diagnosis of rheumatoid arthritis, psoriatic arthritis, axial spondyloarthropathy, juvenile idiopathic arthritis or giant cell arteritis. These bDMARDs were selected based on their initial dosing frequency with an administration more often than once a month. Patients with a bi-weekly (etanercept 2 × 25 mg) dosing schedule were excluded to prevent overlapping assessments in different waves. Participants could withdraw from the study at any time.

### Ethical considerations

The Dutch Medical Research Committee of East Netherlands (METC Oost-Nederland) exempted ethical approval on 3 May 2022 because this study was not subject to the Medical Research Involving Human Subjects Act (file number 2022–13752). This study was approved by the institutional review board of the Sint Maartenskliniek and all participants signed digital informed consent prior to participation. The study was conducted in accordance with the principles of the Declaration of Helsinki.

### Data collection

From October 2022 to January 2023 patients were invited to participate by email through an online Personal Health Record (PHR) named Zorgdoc^®^. Zorgdoc^®^ is also used by patients to order their bDMARDs from the outpatient pharmacy of the Sint Maartenskliniek. After log in patients could read study information and, if patients decided to participate they were asked to sign digital informed consent and to complete a baseline questionnaire in which the date of the upcoming bDMARD injection was registered. Multiple entries were not possible as patients were invited through their PHR.

**Fig. 1:**
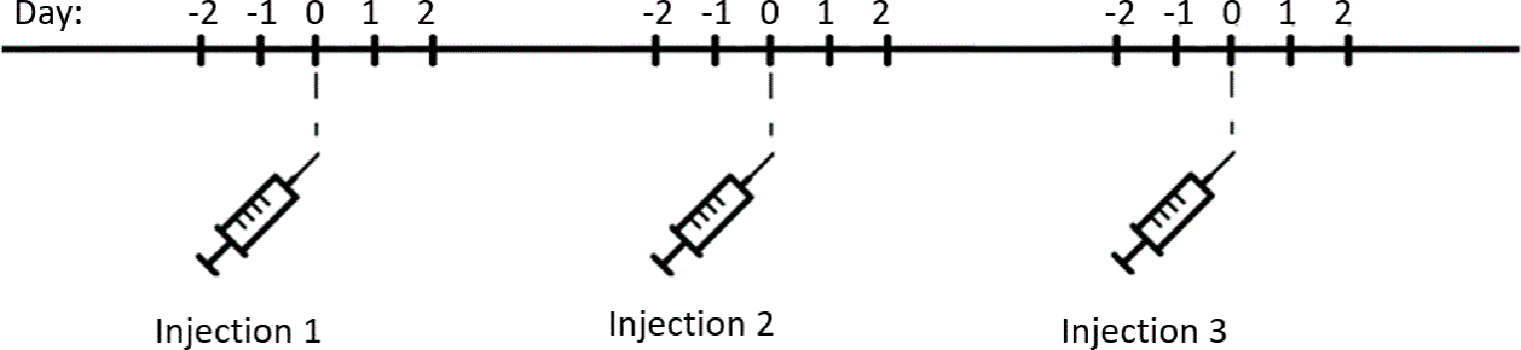
The schedule of ecological momentary assessments in three waves of five days surrounding a bDMARD injection

The upcoming injection date was combined with the dosing schedule to automatically plan the study assessment schedule for each patient in the Zorgdoc^®^ PHR. Participants received a link by email every evening at 6.30 pm for five days in three waves, surrounding three bDMARD injections, to complete one to three web-based ecological momentary assessment items, comprising 15 assessments in total (Fig. 1 and Online Resource 1). The assessment could be completed until 0.00 am that same evening. After completing an assessment, participants could not change the answers. All assessments included an end of day fatigue severity assessment. On day 1 and day 3 of each wave, participants were additionally asked to confirm or correct the planned injection date. On day 5 of the first and second wave, patients were additionally asked to confirm or correct the subsequent injection date. On day 5 of each wave, patients were additionally asked if they experienced any ADRs of the bDMARD. ADRs were coded using Preferred Terms of the Medical Dictionary for Regulatory Activities (MedDRA^®^) following standard pharmacovigilance practice by JvL [18]. The web-based ecological momentary assessments were pretested with five patients following the three-step test-interview [19] and pilot tested by three researchers (JV, VH and JvL) and by three patients.

The following demographic and clinical characteristics were collected from the electronic health records for each participant: age, sex, body mass index, diagnosis, disease activity from 3 months prior to participation until end of study period (DAS28 for rheumatoid arthritis and juvenile idiopathic arthritis, PASDAS for psoriatic arthritis, ASDAS and BASDAI for axial spondyloarthropathy), anti CCP status, rheumatoid factor (RF) status, bDMARD with dosing scheme and its start date, and combination therapy and dosing scheme.

### Measures and data analysis

#### Study sample

The study sample was defined as the number of participants completing at least one pre-dosing and one post-dosing assessment in one wave. Descriptives of study variables were computed as proportions, means with standard deviation (SD) or medians with interquartile ranges (IQRs, 25° − 75° percentile), if appropriate. Data was analysed in R studio (version 4.3.0).

#### Severity of fatigue

Fatigue severity was measured using the fatigue NRS (“Please choose the number that shows your average level of fatigue today”), with 0 “no fatigue” to 10 “totally exhausted”. The overall severity of fatigue was measured by the proportion of patients with clinically relevant fatigue (NRS ≥ 2) and severe fatigue (NRS ≥ 5) in any assessment in any wave [20, 21].

#### Course of fatigue

To describe the course of fatigue surrounding a bDMARD injection, fatigue scores were examined for each wave completed by each patient. The mean of pre-dosing fatigue scores was compared with the mean of post-dosing fatigue scores in the same wave for all waves with at least one completed pre-dosing and post-dosing assessment. For each wave, the change in fatigue from pre-dosing to post-dosing was computed (mean post-dosing minus mean pre-dosing). Thereafter, the proportion of injections categorised into a clinically relevant change (i.e. worsening or improvement) or no clinically relevant change was calculated. An injection with a clinically relevant change was defined as 1.2 for worsening fatigue and − 1.0 for improvement of fatigue, using average cut-off points for minimal clinically important difference (MCID) on the visual analogue scale in patients with rheumatoid arthritis [22].

For all patients completing a pre-dosing and post-dosing assessment in all three waves, patterns in the course of fatigue were investigated. Three distinct patterns were defined: (a) clinically relevant improvement of mean fatigue in at least 2 out of 3 waves, the majority of bDMARD injections, (b) clinically relevant worsening of mean fatigue in at least 2 out of 3 waves, or (c) no clinically relevant change in mean fatigue in at least 2 out of 3 waves. These patterns were also stratified per bDMARD.

#### Self-reported ADRs

The type and frequency of ADRs reported on day 5 of each wave were assessed and described at MedDRA Preferred Term level. The frequency was expressed as the number of reported ADRs, the number of unique ADRs (counting one type of ADR reported by one patient once) and the number and proportion of patients reporting the ADR.

## Results

**Fig. 2:**
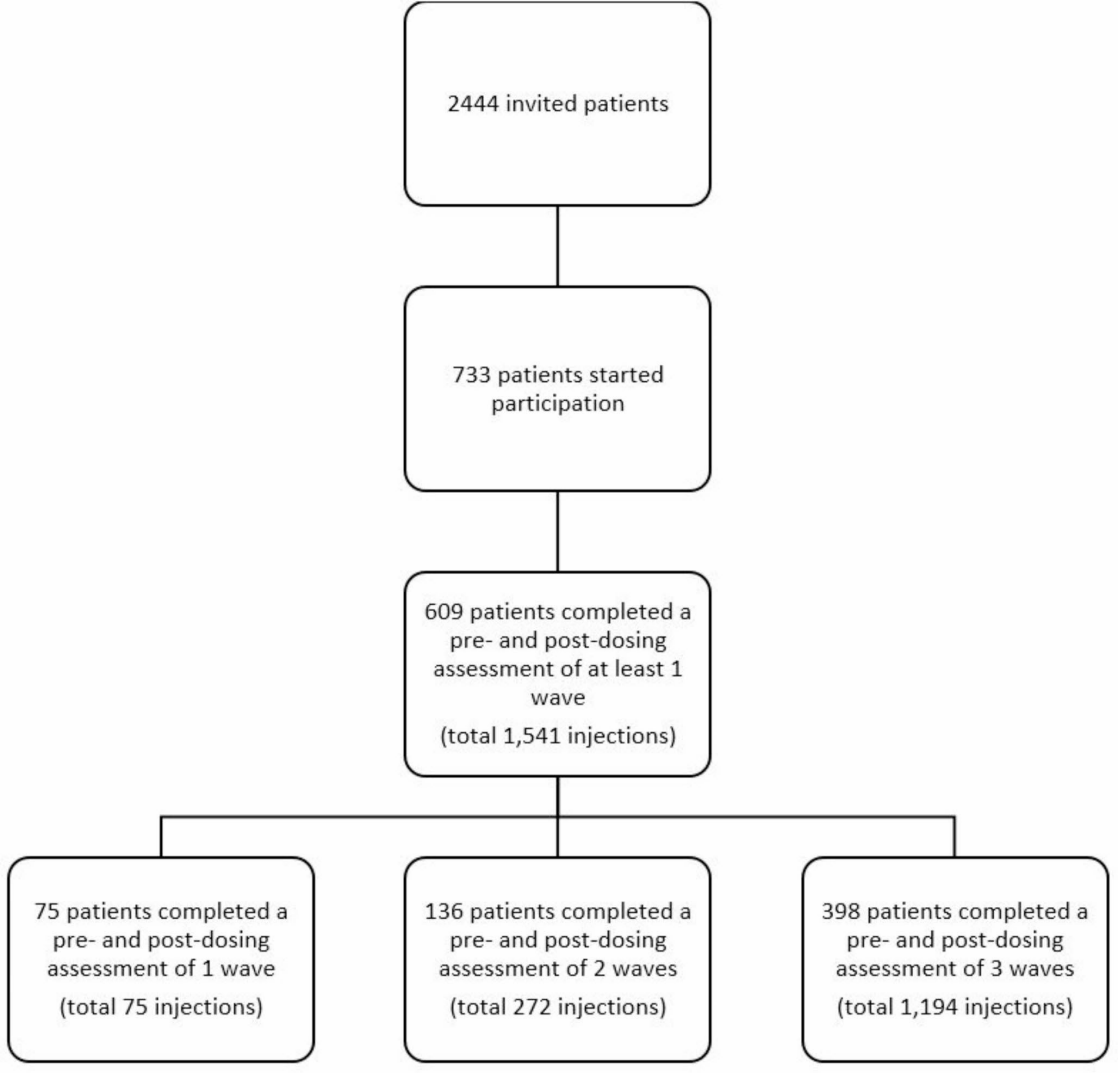
Flowchart of number of invited patients to number of patients completing ecological momentary assessments

Out of 2,444 invited patients, 734 patients (30%) consented to participate, and 609 participants (83% of patients with consent) provided at least one pre-dosing and post-dosing fatigue score of a single wave (Table 1, Online Resource 2 and Fig. 2). Most participants used adalimumab (52%) or etanercept (32%). A total of 398 participants (65%) completed at least one pre-dosing and post-dosing fatigue score of all three waves. At least one pre-dosing and post-dosing fatigue score was completed for 1541 waves in total, which is 84% of the theoretically possible number of 1827 waves completed by 609 patients.

**Table 1:** Baseline characteristics of participants that provided at least one pre-dosing and post-dosing fatigue score of a single wave

	*N* (%)
**Total**	609 (100)
**Female**	384 (63)
**Age, median years (IQR)**	58 (48.5–66)
**BMI, mean (SD)** ^**a**^	27.6 (5)
**Rheumatic disease**	
- Rheumatoid arthritis	343 (56)
- Psoriatic arthritis (mostly peripheral)	144 (24)
- Radiographic axial spondyloarthropathy	96 (16)
- Non-radiographic axial spondyloarthropathy	7 (1)
- Psoriatic arthritis (mostly axial)	11 (2)
- Juvenile idiopathic arthritis	4 (0.6)
- Giant cell arteritis	4 (0.6)
**Disease duration, median years (IQR) at baseline**	8 (3–16)
**Disease activity, median score (IQR) during study period**	
- DAS28 (*n* = 275)	2.2 (1.6–3.0)
- PASDAS (*n* = 76)	3.0 (1.8–3.8)
- ASDAS (*n* = 62)	2.2 (1.7–3.0)
- BASDAI (*n* = 59)	3.8 (2.6–5.5)
**Rheumatoid Factor positivity** ^**b**^ **(% of rheumatoid arthritis patients)**	197 (57)
**Anti-CCP positivity** ^**c**^ **(% of rheumatoid arthritis patients)**	194 (57)
**bDMARD**	
- Adalimumab	318 (52)
- Etanercept	192 (32)
- Tocilizumab	38 (6)
- Abatacept	34 (6)
- Certolizumab	16 (3)
- Sarilumab	11 (2)
**bDMARD treatment duration at baseline in months, median (IQR)**	11 (8–47)
**Other medication prescribed by rheumatologist** ^**d**^	
- NSAID	285 (47)
- Methotrexate	211 (35)
- Corticosteroid	81 (13)
- Leflunomide	47 (8)
- Hydroxychloroquine	37 (6)
- Sulfasalazine	26 (4)

a. BMI was unknown for 225 patients

b. Rheumatoid factor was unknown for 32 rheumatoid arthritis patients

c. Anti-CCP was unknown for 29 rheumatoid arthritis patients

d. Other medication not prescribed by a rheumatologist is shown in Online Resource 2

### Severity of fatigue

Overall mean fatigue severity across all three waves was 4.5 (± SD 2.4) (Online Resource 3). Four hundred and fifty nine of 609 participants (75%) reported clinically relevant fatigue (NRS ≥ 2) in all completed assessments. A total of 477 patients (78%) reported severe fatigue (NRS ≥ 5) in at least one assessment and 145 patients (24%) reported severe fatigue in all completed assessments.

### Course of fatigue (per injection wave)

**Fig. 3:**
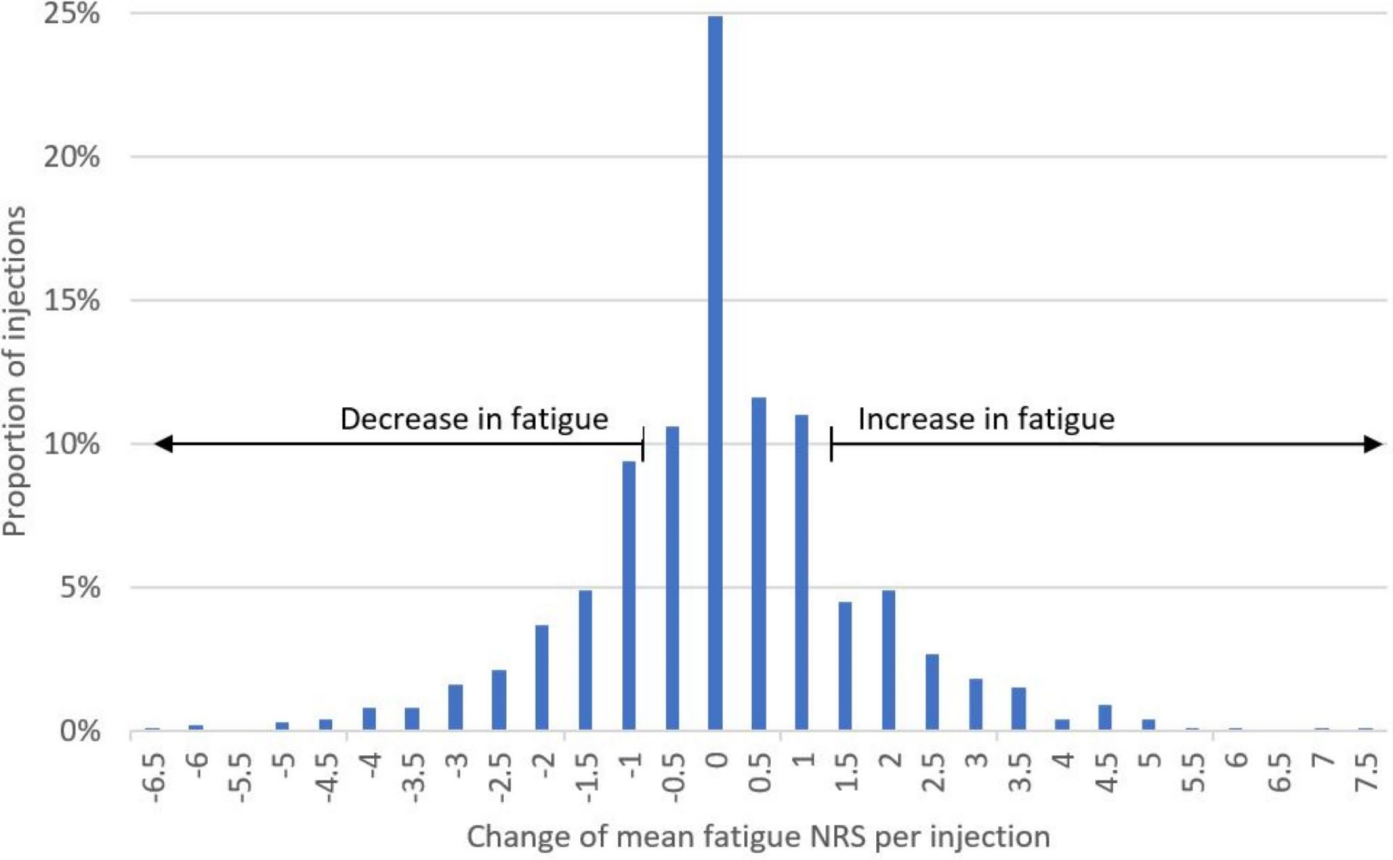
The change of fatigue per injection for all 1,541 injections

Out of 1,541 injections, 17% (267 injections) were followed by worsening in fatigue, while 25% (378 injections) were followed by improvement in fatigue. No clinically relevant change in fatigue scores was observed following 58% (896 injections) of the administered injections (Fig. 3).

### Patterns in course of fatigue (per patient across three waves)

**Fig. 4:**
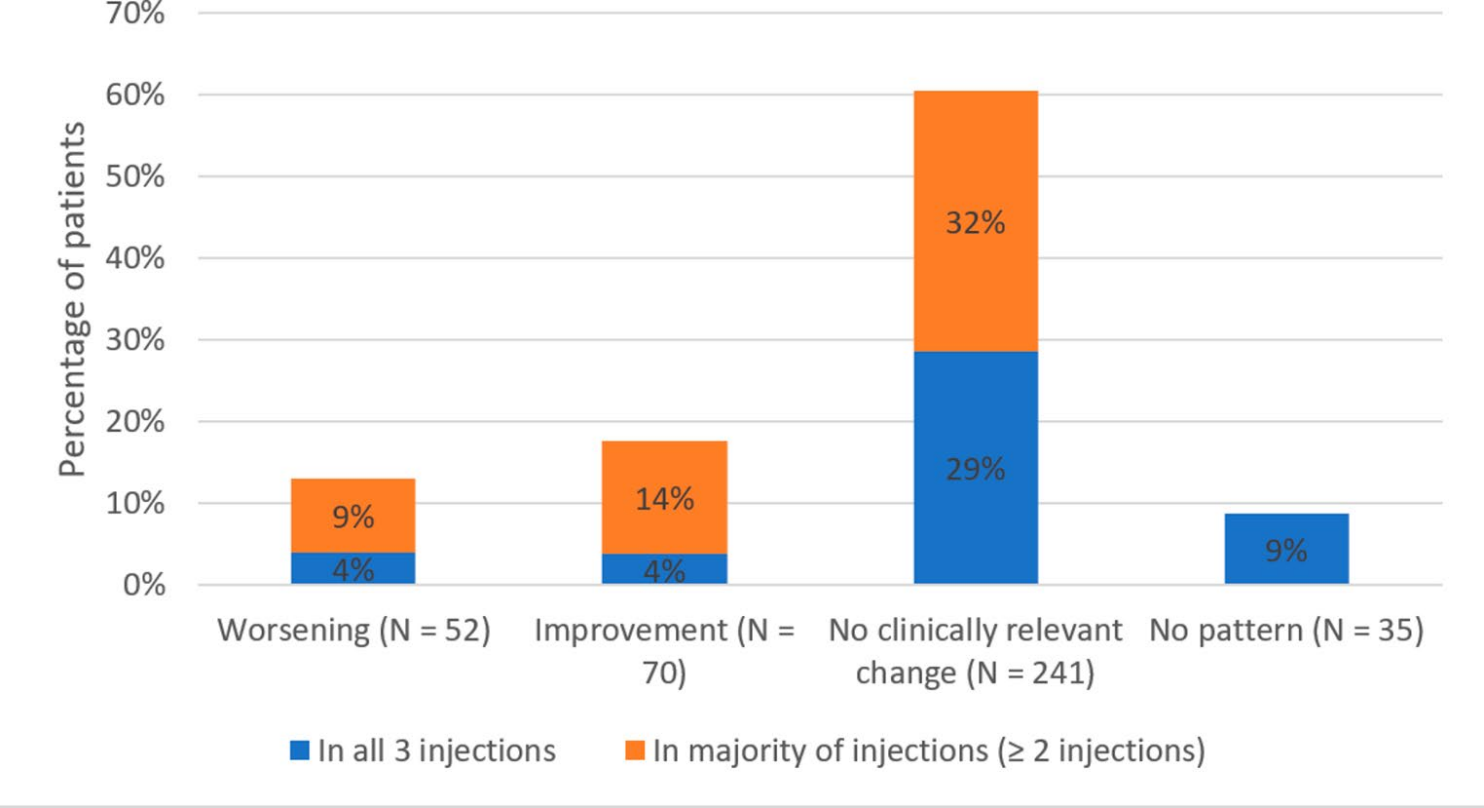
The proportion of patients with a pattern of worsening, improvement or no clinically relevant change of fatigue following bDMARD injection in the majority of injections, out of all patients completing all three waves (*N* = 398)

Of 398 patients completing all three waves, most patients (61%) had no clinically relevant change of fatigue, 13% had a pattern of worsening fatigue and 18% had a pattern of improving fatigue in the majority of injections (Fig. 4). In total 145 patients (36%) had the same consistent pattern of fatigue around all three bDMARD injections. 4% of the patients exhibited a consistent pattern of worsening fatigue following all three injections, while a similar consistent pattern of improvement of fatigue was observed in another 4% of the patients. A post-hoc analysis of 136 patients completing two waves in total showed a similar distribution of a consistent worsening pattern (7%) and consistent pattern of improvement (10%) of fatigue (Online Resource 4).

**Fig. 5:**
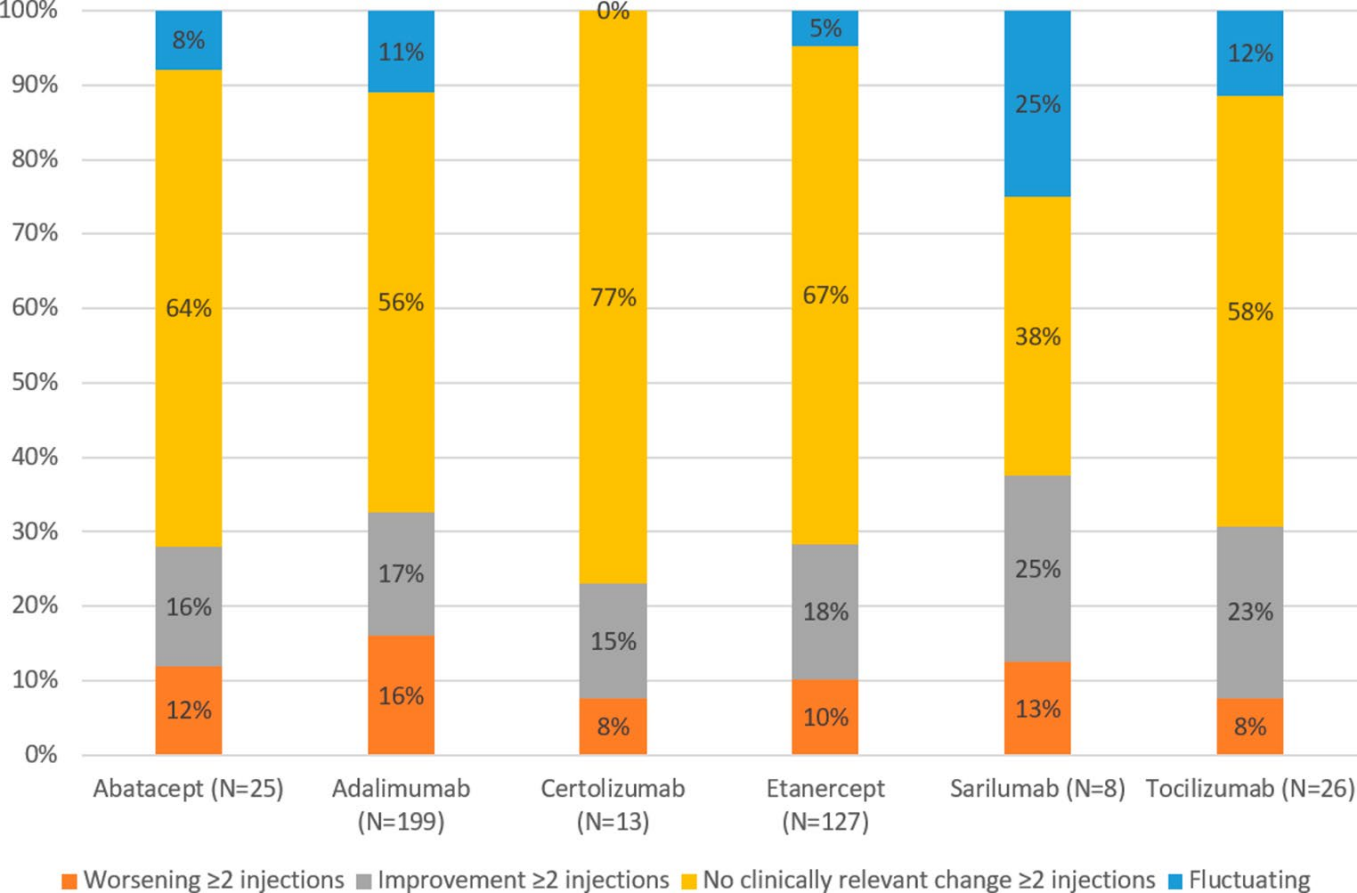
The proportion of patients with a pattern of worsening, improvement or no clinically relevant change of fatigue following bDMARD injection per bDMARD out of all patients completing three waves (*N* = 398)

The distribution of fatigue patterns for each bDMARD were similar to the overall distribution in patterns (Fig. 5). Worsening of fatigue following the majority of injections varied between 8 and 12% and improvement of fatigue varied between 15% and 25% for the various bDMARDs. For each bDMARD, the majority of patients had no clinically relevant change of fatigue after injection, varying between 38% and 77%.

### Self-reported ADRs

Out of 609 patients, 590 patients (97%) completed the last questionnaire (in which they were asked for ADRs) of at least one wave, comprising a total of 1423 injections. 148 patients (25%) reported at least one ADR following 248 injections (17%). In total, 402 ADRs and 320 unique ADRs were reported (Online Resource 5). The mean number of unique reported ADRs was 2.2 (SD 1.3) per patient, varying from 1 to 6 ADRs. The top 5 most reported ADRs was fatigue (reported 76 times by 51 patients, 9%), followed by headache (reported 35 times by 28 patients (5%)), injection site swelling (reported 19 times by 14 patients (2%)), nausea (reported 16 times by 12 patients (2%)) and injection site erythema (reported 16 times by 12 patients (2%)).

Of the 51 patients reporting fatigue as an ADR, a pattern of worsening fatigue following the majority of bDMARD injections was observed in 19 patients (37%), a pattern of no clinically relevant change in fatigue was observed in 11 patients (22%), a pattern of improving fatigue was observed in one patient (2%) and no specific pattern was measured in three patients (6%). The 17 remaining patients reporting fatigue as an ADR did not complete all three waves and patterns were not examined in these patients. However, 13 of these patients completed two waves and five of these patients had consistent worsening of fatigue following bDMARD injection in both waves.

## Discussion

This ecological momentary assessment study demonstrated the severity, course and specific patterns in course of fatigue surrounding subcutaneous bDMARD injection in IRD patients. In most patients, fatigue remained stable around bDMARD injection and more or less similar proportions of patients had an improving (18%) or worsening (13%) pattern following the majority of administered bDMARD injections administered during the study period. Overall fatigue severity was clinically relevant, addressing the significance of fatigue in this patient population in general. In addition to the quantified severity and course of fatigue, fatigue was the most frequently reported ADR and was reported by 9% of patients which comprised 37% (19/52) of the patients with a pattern of worsening fatigue following bDMARD injection.

As far as we know, individual fatigue patterns around bDMARD injection have not been quantified before. A study has demonstrated post-dosing fatigue patterns with methotrexate by quantifying patient-reported nausea and fatigue before and after administration [14]. In addition, distinct longitudinal fatigue trajectories have been identified before, despite minimal average changes on a group level [20]. Even though the cause of fatigue is known to be multifactorial and the pharmacological mechanism of bDMARD-associated fatigue remains unclear, fatigue is labelled as an ADR in the SmPC of various bDMARDs. We identified a similar proportion of patients reporting fatigue as an ADR in this study (9%) compared to the previous cohort event monitoring study (8%) [16]. In the previous study, 4% described fatigue as a postdosing reaction which is similar to 3% (19/609) of patients with a worsening fatigue pattern following injection also reporting fatigue as an ADR in this study. Nevertheless, reporting methods of both studies differed and the current study did not aim to demonstrate an association between bDMARDs and fatigue. Furthermore, we demonstrate similar proportions of patients with improving and worsening fatigue.

Several explanations for different fatigue patterns around bDMARD injection can be considered. A pharmacological basis for fluctuations of fatigue around injection seems unlikely since time to peak drug concentrations of the various included bDMARDs do not align with the ecological momentary assessments two days after injection. Fatigue is prevalent in healthy people as well as in patients with chronic diseases and various individual day to day fluctuations of fatigue have been demonstrated [23, 24]. Fatigue fluctuations around injection could be related to patient expectations considering the effect of the injection. Patients know that they are injecting the drug, and this knowledge can be associated with positive or negative cognitions and emotions, leading to experiencing improvement or worsening of fatigue as a placebo or nocebo effect respectively [25]. Another explanation is that fatigue changes around use of medication occur by chance. Furthermore, studies investigating the effect of bDMARDs on fatigue are limited and a study evaluating the effect of biologic interventions on fatigue by pooling results of 32 studies concerning 9,946 bDMARD users demonstrated fatigue improvements after bDMARD initiation [15]. However, that study assessed fatigue on a group level and in active IRD patients, and did not assess individual fluctuations specifically around injection. As the complexity of many factors involved in fatigue in IRD patients is well known, understanding fatigue fluctuations remains challenging.

As patients may experience different patterns, attention to individual fatigue patterns is recommended. Patients who mention a specific fatigue pattern in clinical practice could benefit from their healthcare professional’s support in coping with fatigue. These patients could be informed that most patients do not experience specific patterns but that improving as well as worsening patterns have been reported. Overall, providing profound information about fatigue is important for all IRD patients in decreasing the burden of fatigue [17].

This study has some limitations. This was an exploratory study and to minimalize the administration burden for participations, fatigue was measured only in patients on open label subcutaneous bDMARDS, and for a short period of five days around injection. Therefore fluctuations in fatigue at times other than surrounding injection could not be investigated. Other variables potentially associated with fatigue such as sleep disturbances, pain, mood, disease perception and daily activities were not measured and could therefore not be taken into account [4, 9, 17]. Also, patients could only complete each ecological momentary assessment in the evening and not all assessments were completed which led to missing data. Nonetheless, the high proportion of patients completing at least one pre-dosing and one post-dosing assessment out of the total number of patients starting participation (83%, 609/734) addresses the dedication of these patients and the importance of fatigue in this population.

As this study is the first to explore and identify fatigue fluctuations surrounding bDMARD injection, future research should focus on how to manage these effects in clinical practice. In addition, other factors strongly associated with fatigue, such as obesity, depression, poor sleep and pain should be investigated in relation to fatigue patterns [4]. Since this is the first study quantifying fatigue fluctuations surrounding bDMARD administration and this study solely focused on IRD patients, it is unknown if similar patterns would be identified in other patient populations using bDMARDs, such as in dermatology or gastroenterology.

## Conclusion

Previous research indicated that patients sometimes experience fatigue as an ADR of bDMARDs, specifically recurring around bDMARD injection. This study demonstrated that some patients experience consistent patterns in the course of fatigue following bDMARD injection and identified similar proportions of patients with a worsening and improving fatigue pattern. Since fatigue is a major issue for IRD patients, recognizing individual patterns and informing patients properly can contribute to fatigue management in clinical practice.

## Electronic supplementary material

Below is the link to the electronic supplementary material.

Supplementary Material 1

## Data Availability

The data supporting this study’s findings are available upon reasonable request from the corresponding author.
