# Supplementary material for: Fatigue patterns surrounding biologic disease-modifying antirheumatic drug injection in patients with an inflammatory rheumatic disease: an ecological momentary assessment study
Source: Rheumatol Int. 2025 Jan 11;45(1):24. doi: 10.1007/s00296-024-05779-y (PMC11724786; doi:10.1007/s00296-024-05779-y)
Supplement: Supplementary file 1 — Supplementary Material 1 [file 296_2024_5779_MOESM1_ESM.pdf]

Supplementary material

# Fatigue patterns surrounding biologic disease-modifying antirheumatic drug injection in patients with an inflammatory rheumatic disease: an ecological momentary assessment study

Jette van Lint, Johanna Vriezekolk, Naomi Jessurun, Alfons den Broeder, Bart van den Bemt, Victor Huiskes

Journal name: Rheumatology International

Corresponding author:

Jette A van Lint
E-mail: jette.vanlint@radboudumc.nl
Netherlands Pharmacovigilance Centre Lareb
Goudsbloemvallei 7
5237 MH, 's-Hertogenbosch
T: +31 73 6469700

**Supplementary material:**

- Online Resource 1. Baseline questionnaire and ecological momentary assessments (Translated from Dutch)
- Online Resource2: Table S1. Medication used by participants not prescribed by a rheumatologist as registered in electronic health records classified according to the World Health Organisation Anatomical Therapeutic Chemical index.
- Online Resource 3: Figure S1. The overall mean fatigue NRS scores and standard deviation of three waves, surrounding three bDMARD injections.
- Online Resource 4: Figure S2. The proportion of patients with worsening, improvement or no clinically relevant change of fatigue following bDMARD injection of patients completing two waves in total.
- Online Resource 5:Table S2. Reported adverse drug reactions two days after bDMARD injection according to Preferred Terms of the Medical Dictionary for Regulatory Activities.

**Online Resource 1. Ecological momentary assessments per wave (text and questions translated from Dutch)**

**Baseline**

What day is the next injection of your biologic planned? [Calendar]

- *In case a date later than two days after completing the question is chosen*: You will receive the first questionnaire on (*date*). This is two days before your injection.
- *In case a date within two days after completing the question is chosen*: Your next injection is tomorrow or the day after tomorrow. That does not give us enough time to send you the first questionnaire (two days before injection). What day will you inject the next dose ? [Calendar without upcoming 2 days] → You will receive the first questionnaire on (*date*). This is two days before your next injection.

**Day 1**

*1.* Is the next injection of your biologic the day after tomorrow (*day month*)? [Yes/No]

→ If yes:

a. Please choose the number that shows your average level of fatigue **today**:

   No fatigue 0 1 2 3 4 5 6 7 8 9 10 Totally exhausted

→ If no:

b. Will you inject your biologic another day and if yes, when?
   i. **Yes** → [Calendar]
   - *If date at least 2 days later is chosen:* You will receive a new questionnaire on (*date)*. This is two days before your next injection.
   - *If date within 2 days is chosen*: Your next injection is tomorrow or the day after tomorrow. That does not give us enough time to send you the first questionnaire (two days before injection). What day will you inject the next dose ? [Calendar without upcoming 2 days] → You will receive the first questionnaire on (*date*). This is two days before your next injection.

   ii. **I am not sure yet** → As the date of your next injection is unknown, we now presume (*date*) *(= 7 days after planned injection date of current wave*). This is one week later. You will receive the next questionnaire on (*date*) (*date = 7 days later / 5 days after planned injection date of current wave*]. If the presumed injection date is not correct, you can specify the new injection date in that questionnaire.

   iii. **No, I no longer use the biologic** → As you no longer use the biologic, your participation in this study will be discontinued. Thank you for participating.

**Day 2**

1. Please choose the number that shows your average level of fatigue **today**:

   No fatigue 0 1 2 3 4 5 6 7 8 9 10 Totally exhausted

**Day 3**

1. Did you inject your biologic today or will you inject your biologic today? [Yes/No]

→ *If No, assessments in this wave stop and questions for verifying the next:*

a. Will you inject your biologic another time and if yes, when?
   i. **Yes** → [Calendar]
      - *If date at least 2 days later is chosen:* You will receive the next questionnaire on *(Date two days before injection date)*
      - *If date within 2 days is chosen:* Your next injection is tomorrow or the day after tomorrow. That does not give us enough time to send you the first questionnaire (two days before injection). What day will you inject the next dose ? [Calendar without upcoming 2 days] → You will receive a new questionnaire on (*date*). This is two days before your next injection.

   ii. **I am not sure yet** → As the date of your next injection is unknown, we now presume (*date*) *(= 7 days later / 7 days after planned injection date of current wave*). This is one week later. You will receive the next questionnaire on (*date*) (*date = 5 days later / 5 days after planned injection date of current wave*). If the presumed injection date is not correct, you can specify the new injection date in that questionnaire.

   iii. **No, I no longer use the biologic** → As you no longer use the biologic, your participation in this study will be discontinued. Thank you for participating.

→ *If yes*:

2. What time did you or will you inject your biologic? *[Time: 00:00 to 23:59]*
3. Please choose the number that shows your average level of fatigue **today**:

   No fatigue 0 1 2 3 4 5 6 7 8 9 10 Totally exhausted

**Day 4**

1. Please choose the number that shows your average level of fatigue **today**:

   No fatigue 0 1 2 3 4 5 6 7 8 9 10 Totally exhausted

**Day 5 of first and second wave**

1. Please choose the number that shows your average level of fatigue **today**:

   No fatigue 0 1 2 3 4 5 6 7 8 9 10 Totally exhausted

2. Did you experience a side effect of your biologic in the last two days? You may also describe your complaint if you are not sure your complaint is a side effect.
   - Yes, namely: [*Free text*]
   - No
3. Is it correct that the next injection of your biologic is on [*date = suggested injection date based on personal dosing frequency*]? [Yes/No]
   → *If yes*: You will receive the next questionnaire on (*date*). This is two days before your next injection.

   → If no:

a. Will you inject your biologic another time and if yes, when?
   - **Yes** → [Calendar]
   - **I am not sure yet** → As the date of your next injection is unknown, we now presume (*date*) *(= 7 days after planned injection date*). This is one week later. You will receive the next questionnaire on (*date*) (*date = 5 days after planned injection date)*. If that injection date is not correct, you can specify the new injection date in that questionnaire.
   - **No, I no longer use the biologic** → As you no longer use the biologic, your participation in this study will be discontinued. Thank you for participating.

**Day 5 of third wave**

1. Please choose the number that shows your average level of fatigue **today**:

   No fatigue 0 1 2 3 4 5 6 7 8 9 10 Totally exhausted

2. Did you experience a side effect of your biologic in the last two days? You may also describe your complaint if you are not sure your complaint is a side effect.
   - Yes, namely: [*Free text*]
   - No

**Online Resource 2**

**Supplementary Table S1. Medication used by participants not prescribed by a rheumatologist as registered in electronic health records classified according to the World Health Organisation Anatomical Therapeutic Chemical index.**

| **Medication group** | **Anatomical Therapeutic Chemical code** | **N (%)** |
|---|---|---|
| Drugs for acid related disorders | A02 | 317 (52) |
| Anti-emetics | A04 | 10 (2) |
| Drugs for constipation | A06 | 61 (10) |
| Drugs used in diabetes | A10 | 35 (6) |
| Antithrombotic agents | B01 | 90 (15) |
| Cardiovascular system drugs | C | 255 (42) |
| Thyroid therapy | H03 | 25 (4) |
| Drugs for treatment of bone diseases | M05 | 42 (7) |
| Analgesics | N02 | 209 (34) |
| Antiepileptics | N03 | 11 (2) |
| Psycholeptics | N05 | 52 (9) |
| Antidepressants | N06A | 52 (9) |
| Medication for obstructive airway diseases | R03 | 74 (12) |
| Antihistamines for systemic use | R06 | 74 (12) |
| Other | - | 437 (72) |

**Online Resource 3**

**Supplementary Figure S1. The overall mean fatigue NRS scores and standard deviation of three waves, surrounding three bDMARD injections.**

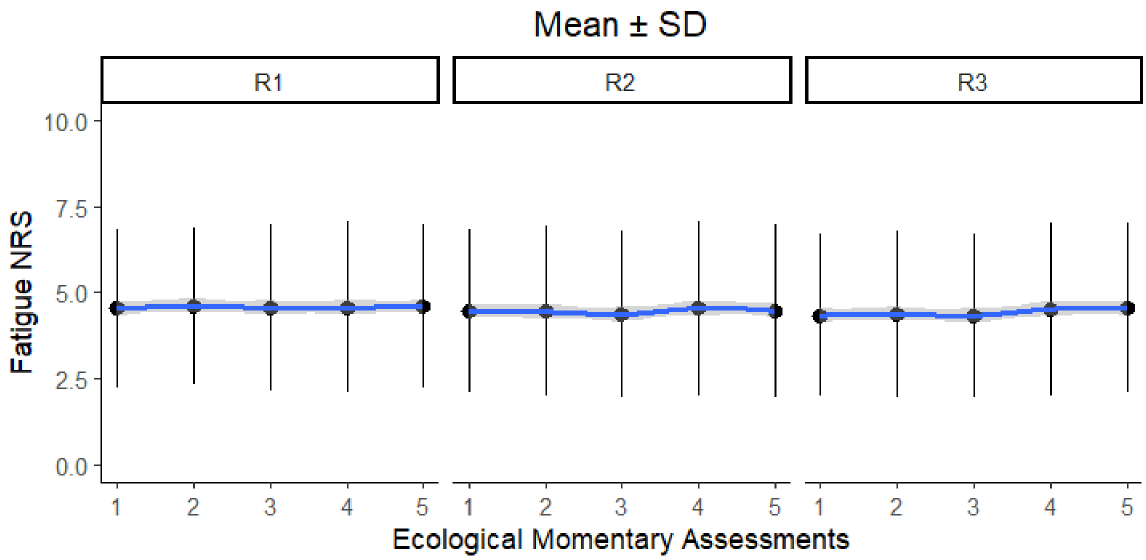

**Online Resource 4**

**Supplementary Figure S2. The proportion of patients with worsening, improvement or no clinically relevant change of fatigue following bDMARD injection of patients completing two waves in total (N=136).**

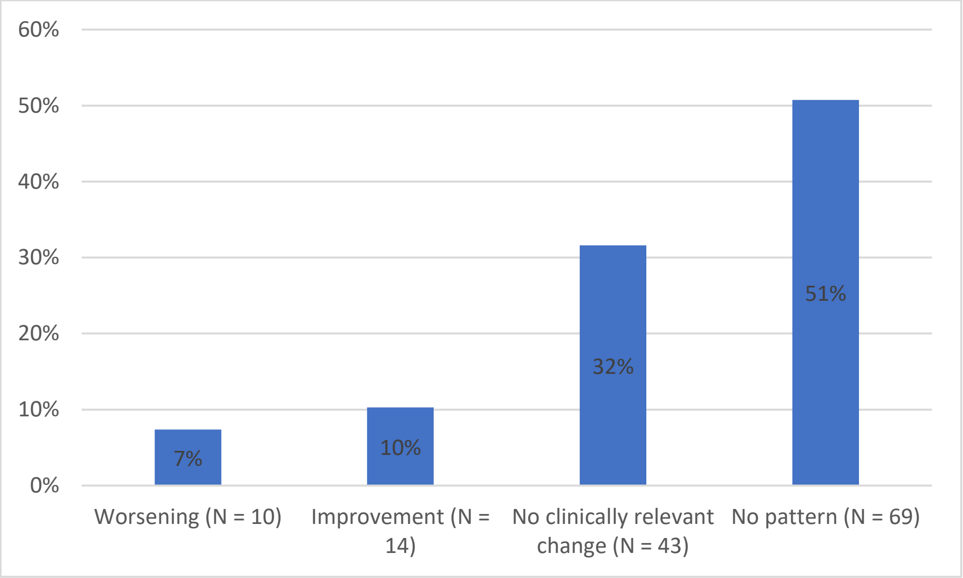

**Online Resource 5**

**Supplementary Table S2. Reported adverse drug reactions two days after bDMARD injection according to Preferred Terms of the Medical Dictionary for Regulatory Activities**

| **Adverse drug reaction (MedDRA Preferred Terms)** | **Number of reported ADRs (Total 402)** | **Number of patients reporting the ADR (N= 590) N (%)** |
|---|---|---|
| **Total** | **402** | **148 (25)** |
| Fatigue | 76 | 51 (9) |
| Headache | 35 | 28 (5) |
| Injection site swelling | 19 | 14 (2) |
| Nausea | 16 | 12 (2) |
| Injection site erythema | 16 | 12 (2) |
| Pruritus | 15 | 10 (2) |
| Injection site pruritus | 14 | 11 (2) |
| Dizziness | 9 | 6 (1) |
| Abdominal discomfort | 9 | 7 (1) |
| Malaise | 8 | 7 (1) |
| Injection site pain | 7 | 7 (1) |
| Nasopharyngitis | 6 | 4 (0.7) |
| Asthenia | 6 | 6 (1) |
| Sleep disorder | 6 | 6 (1) |
| Rash | 6 | 5 (0.8) |
| Myalgia | 6 | 6 (1) |
| Arthralgia | 6 | 5 (0.8) |
| Diarrhoea | 6 | 5 (0.8) |
| Influenza like illness | 6 | 4 (0.7) |
| Injection site haematoma | 5 | 5 (0.7) |
| Pain in extremity | 5 | 4 (0.7) |
| Somnolence | 5 | 4 (0.7) |
| Emotional disorder | 4 | 3 (0.5) |
| Erythema | 4 | 3 (0.5) |
| Back pain | 4 | 4 (0.7) |
| Alopecia | 3 | 2 (0.3) |
| Pain | 3 | 2 (0.3) |
| Dry skin | 3 | 2 (0.3) |
| Abdominal pain | 3 | 3 (0.5) |
| Inflammation | 3 | 2 (0.3) |
| Injection site reaction | 3 | 2 (0.3) |
| Restlessness | 3 | 2 (0.3) |
| Insomnia | 3 | 3 (0.5) |
| Musculoskeletal stiffness | 3 | 3 (0.5) |
| Injection site warmth | 3 | 2 (0.3) |
| Visual impairment | 2 | 2 (0.3) |
| Odynophagia | 2 | 1 (0.2) |

| | | |
|---|---|---|
| Nasal congestion | 2 | 2 (0.3) |
| Hypoaesthesia | 2 | 2 (0.3) |
| Fungal infection | 2 | 1 (0.2) |
| Dyspnoea | 2 | 2 (0.3) |
| Weight increased | 2 | 1 (0.2) |
| Injection site rash | 2 | 2 (0.3) |
| Depressed mood | 2 | 1 (0.2) |
| Dry mouth | 2 | 1 (0.2) |
| Oral herpes | 2 | 2 (0.3) |
| Listless | 2 | 2 (0.3) |
| Dry eyes | 2 | 2 (0.3) |
| Chills | 2 | 2 (0.3) |
| Migraine | 2 | 2 (0.3) |
| Muscle spasms | 2 | 2 (0.3) |
| Drowsiness | 1 | 1 (0.2) |
| Oral pain | 1 | 1 (0.2) |
| Haematoma | 1 | 1 (0.2) |
| Head discomfort | 1 | 1 (0.2) |
| Palpitations | 1 | 1 (0.2) |
| Aphthous ulcer | 1 | 1 (0.2) |
| Skin reaction | 1 | 1 (0.2) |
| Chest pain | 1 | 1 (0.2) |
| Injection related reaction | 1 | 1 (0.2) |
| Ear pain | 1 | 1 (0.2) |
| Flatulence | 1 | 1 (0.2) |
| Feeling abnormal | 1 | 1 (0.2) |
| Pharyngitis | 1 | 1 (0.2) |
| Irritability | 1 | 1 (0.2) |
| Restless legs syndrome | 1 | 1 (0.2) |
| Joint stiffness | 1 | 1 (0.2) |
| Gait disturbance | 1 | 1 (0.2) |
| Hepatic pain | 1 | 1 (0.2) |
| Night sweats | 1 | 1 (0.2) |
| Glossitis | 1 | 1 (0.2) |
| Abscess | 1 | 1 (0.2) |
| Infection | 1 | 1 (0.2) |
| Oropharyngeal pain | 1 | 1 (0.2) |
| Infection susceptibility increased | 1 | 1 (0.2) |
| Fluid retention | 1 | 1 (0.2) |
| General physical health deterioration | 1 | 1 (0.2) |
| Paraesthesia | 1 | 1 (0.2) |
| Feeling cold | 1 | 1 (0.2) |
| Flushing | 1 | 1 (0.2) |
| Cough | 1 | 1 (0.2) |
| Rash macular | 1 | 1 (0.2) |

| | | |
|---|---|---|
| Nail bed inflammation | 1 | 1 (0.2) |
| Energy increased | 1 | 1 (0.2) |
| Dysphonia | 1 | 1 (0.2) |
| Injection site hypersensitivity | 1 | 1 (0.2) |
| Arthritis | 1 | 1 (0.2) |
| Vision blurred | 1 | 1 (0.2) |
| Adverse drug reaction | 1 | 1 (0.2) |
| Bloated feeling | 1 | 1 (0.2) |
| Neck pain | 1 | 1 (0.2) |
| Musculoskeletal discomfort | 1 | 1 (0.2) |
